# Phenotypic Evaluation of Nucleoside Analogues against *Trypanosoma cruzi* Infection: In Vitro and In Vivo Approaches

**DOI:** 10.3390/molecules27228087

**Published:** 2022-11-21

**Authors:** Ludmila F. de A. Fiuza, Denise G. J. Batista, Roberson D. Girão, Fabian Hulpia, Paula Finamore-Araújo, Mustafa M. Aldfer, Ehab Kotb Elmahallawy, Harry P. De Koning, Otacílio Moreira, Serge Van Calenbergh, Maria de Nazaré C. Soeiro

**Affiliations:** 1 Laboratório de Biologia Celular, Instituto Oswaldo Cruz, Fundação Oswaldo Cruz, Av. Brasil, 4365 Manguinhos, Rio de Janeiro 21040-360, Brazil; 2 Laboratory for Medicinal Chemistry (Campus Heymans), Ghent University, Ottergemsesteenweg 460, B-9000 Ghent, Belgium; 3 Laboratório de Virologia Molecular, Instituto Oswaldo Cruz, Fundação Oswaldo Cruz, Rio de Janeiro, Rio de Janeiro 20000-000, Brazil; 4 School of Infection and Immunity, College of Medical, Veterinary and Life Sciences, University of Glasgow, Glasgow 62694, UK; 5 Department of Zoonoses, Faculty of Veterinary Medicine, Sohag University, Sohag 82524, Egypt

**Keywords:** Chagas disease, *Trypanosoma cruzi*, experimental chemotherapy, nucleoside derivatives, thymidine transporter

## Abstract

Chagas disease, caused by *Trypanosoma cruzi* (*T. cruzi*), is a serious public health problem. Current treatment is restricted to two drugs, benznidazole and nifurtimox, displaying serious efficacy and safety drawbacks. Nucleoside analogues represent a promising alternative as protozoans do not biosynthesize purines and rely on purine salvage from the hosts. Protozoan transporters often present different substrate specificities from mammalian transporters, justifying the exploration of nucleoside analogues as therapeutic agents. Previous reports identified nucleosides with potent trypanocidal activity; therefore, two 7-derivatized tubercidins (FH11706, FH10714) and a 3′-deoxytubercidin (FH8513) were assayed against *T. cruzi*. They were highly potent and selective, and the uptake of the tubercidin analogues appeared to be mediated by the nucleoside transporter TcrNT2. At 10 μM, the analogues reduced parasitemia >90% in 2D and 3D cardiac cultures. The washout assays showed that FH10714 sterilized the infected cultures. Given orally, the compounds did not induce noticeable mouse toxicity (50 mg/kg), suppressed the parasitemia of *T. cruzi*-infected Swiss mice (25 mg/kg, 5 days) and presented DNA amplification below the limit of detection. These findings justify further studies with longer treatment regimens, as well as evaluations in combination with nitro drugs, aiming to identify more effective and safer therapies for Chagas disease.

## 1. Introduction

Chagas disease (CD), a tropical neglected disease, is caused by the hemoflagellate parasite *Trypanosoma cruzi* (*T. cruzi*) [1,2]. Due to population mobility from Latin America, CD also represents a public health issue in non-endemic areas, affecting more than six million people worldwide [3,4,5]. CD transmission involves vectorial transmission by triatomine bugs, blood transfusion, organ transplantation, vertical transmission and oral routes by the ingestion of parasite-contaminated food and drink [6,7,8]. CD displays two sequential clinical phases: the acute and the chronic phases. The acute phase is mainly asymptomatic/oligosymptomatic, being characterized by positive parasitemia [9,10]. In immune competent individuals, the parasitism is controlled (but not eliminated), and the disease progresses to the second, chronic stage, characterized by sub-patent and intermittent parasitism. In most cases, the infected individuals remain asymptomatic, but 30–40% develop serious clinical alterations due to cardiac and/or digestive disorders [2,11].

CD treatment is restricted to two old oral drugs that are in clinical use for more than 5 decades, i.e., the nitro-heterocyclic derivatives nifurtimox and benznidazole (Bz). Both have severe limitations due to their limited efficacy, particularly in the chronic stage, the long duration of treatment and the occurrence of resistant parasitic strains. Moreover, the unpleasant side effects often result in treatment dropout [12,13,14]. New, safe, effective and short-course therapies for CD are therefore urgently awaited.

Since the parasites are purine auxotrophic and thus dependent on the acquisition of purine nucleobases and nucleosides from host cells, they are very vulnerable to inhibitors of purine salvage pathways or subversive substrates [15]. Recently, the cloning and characterization of *T. cruzi* purine and pyrimidine transporters of the Equilibrative Nucleoside Transporter (ENT) family has reported [16,17], and amastigotes display the highest transcriptional levels for all four ENT genes [16]. The high substrate affinity of these ENTs and their different substrate affinities from mammalian transporters represent an adaptation to their intracellular localization [15,16] and reinforces studies using nucleoside and/or nucleobase analogues as potential therapeutic weapons. Indeed, the nucleoside antibiotic tubercidin is well known to exert potent activity against *T. cruzi*, and experimentally induced resistance to this drug was linked to nucleoside transport deficiencies [18]. However, tubercidin was too toxic to use against Chagas disease, and safer analogues need to be identified while retaining the anti-parasite activity. In this context, the synthesis and phenotypic analysis of a series of modified nucleosides have been performed. Some analogues with promising activity were identified, specially towards the intracellular forms of the parasite but up to now, failed to achieve full in vivo sterilization [19,20,21,22]. The activity of antiprotozoal nucleoside analogs depends on the presence of suitable nucleoside transporters in the target cells [23,24,25], and thus the differences in the substrate recognition between protozoan and human transporters can be exploited to increase specificity [26].

**Figure 1:**
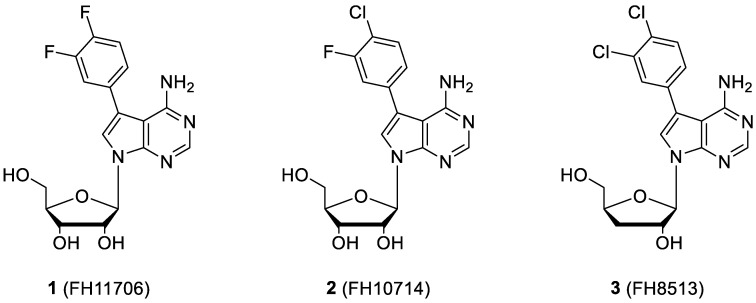
Structural formulae of the compounds under study. Internal codes between brackets.

In the present study, three analogues (two 7-derivatised tubercidin, **1** (internal code **FH11706**) and **2** (**FH10714**) and one 3′-deoxytubercidin, **3** (**FH8513**)) were further assayed (Figure 1) on distinct parasite forms and strains in 2- and 3D cell cultures. Their activity was further explored in a mouse model of acute *T. cruzi* infection. Also, a *T. cruzi* nucleoside transporter, TcrNT2, was shown to mediate the uptake of tubercidin and its 7-substituted analogs.

## 2. Results

### 2.1. In Vitro Activity and Selectivity against Intracellular Forms of T.cruzi (Tulahuen Strain)

The 7-(3,4-Difluorophenyl)tubercidin (**1**, **FH11706**), 7-(3-fluoro,4-chlorophenyl)tubercidin (**2**, **FH10714**) and 7-(3,4-dichlorophenyl)-3′-deoxytubercidin (**3**, **FH8513**) gave excellent activities against intracellular forms of *T. cruzi* (the Tulahuen strain), with EC_50_ values ranging from 0.2 to 1.11 µM. Compound **3 (FH8513)** displayed a 10-fold higher potency as compared to Bz. Compounds **2** (**FH10714**) and **3 (FH8513)** also showed encouraging EC_90_ values in the low µM range. The derivatives exerted a low to moderate toxicity against L929 cells, resulting in high selectivity against (SI > 100), a condition sine qua non for valuable hits (Table 1).

### 2.2. In Vitro Activity against Bloodstream Forms of T.cruzi (Y strain)

Next, the compounds were screened against the other relevant form of human infection, the bloodstream trypomastigotes (BT). The analogues were active against BT, and compound **3** (**FH8513**) displayed EC_50_ and EC_90_ values comparable to those of Bz (Table 2).

### 2.3. In Vitro Cardiotoxicity 

To assess potential cardiotoxicity, uninfected cardiomyoblasts (H9C2) monolayers were inspected after 24 h of drug incubation. **FH11706**, **FH10714** and Bz were not cardiotoxic up to 300 µM, while **FH8513** gave an IC_50_ value of 267.5 µM (Table 2). To further investigate cardiotoxic events after longer periods of drug exposure, 2- and 3D H9C2 cultures were incubated for seven days. Pentamidine (Pt) and Bz were used as positive and negative controls, respectively (Table 3). The nucleoside analogues were toxic on the 2D cultures after seven days of drug incubation, resulting in IC_50_ values ranging from 18.57–47.71 µM. On the other hand, FH10714 and FH8513 showed mild measurable toxicity (IC50 values 180 and 199 µM, respectively) towards the organoids.

### 2.4. In Vitro Activity against Intracellular Forms T. cruzi (Y strain) in 2D and 3D H9C2 Cultures

Next, the derivatives were further investigated during the infection of both 2D and 3D H9C2 cultures incubated for seven days with 10 µM of each compound. This concentration represents the EC_90_ value of Bz against the intracellular forms of *T. cruzi* [27] and is recommend as a first filter in a screening cascade of novel hits for Chagas disease [28]. The parasite load was determined by both light microscopy and qPCR. A high reduction in the parasitism of the 2D and 3D cultures was observed for all tested compounds (83.8–100%) as determined by the light microscopy evaluation. qPCR analysis achieved comparable results, confirming the promising anti-*T. cruzi* activity of the derivatives in both the monolayers and microtissues (Table 4).

### 2.5. In Vitro Analysis of Derivatives Uptake by TcrNT2 Thymidine Transporter

**Figure 2:**
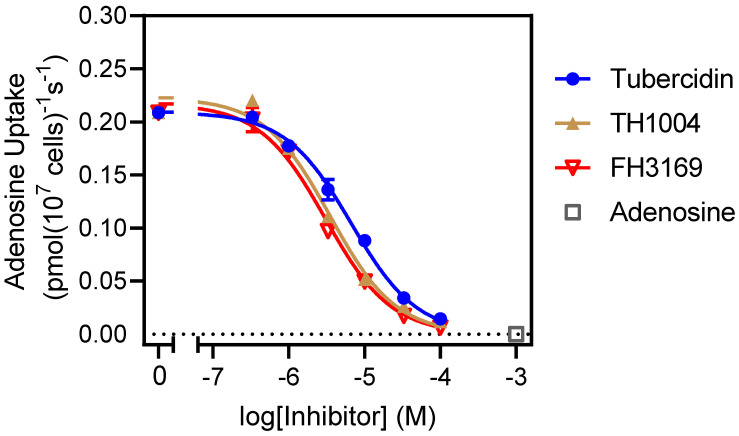
Uptake of 50 nM [^3^H]-adenosine by LmexNT1.1 expressed in *L. mexicana* promastigotes from which both original alleles of LmexNT1.1/NT1.2 and of LmexNT2 had been deleted by CRISPR/cas9. Incubation time was 10 s, well within the linear phase of uptake [30]. Symbols represent averages of triplicate determinations, and error bars indicate ± SEM when not shown error bars fall within the symbol. The graph shown is a single experiment in triplicate, representative of three identical, independent repeats.

To investigate whether the TcrNT2 thymidine transporter might be involved in the uptake of the nucleoside analogues, the transporter was expressed in a *Leishmania mexicana* strain from which LmexNT1.1 and LmexNT1.2 had been deleted (Lmex^ΔNT1^; [17]). As previously shown, the deletion of this NT1.1/1.2 locus creates a virtual *null* background for the uptake of adenosine and pyrimidine nucleosides in *Leishmania* spp. [17,29]. The *T. cruzi* thymidine transporter had already been implicated in the transport of, and resistance to, tubercidin [17,18] and expressing it in this *Leishmania* strain should make a sensitive test for uptake of tubercidin analogues. Accordingly, tubercidin, **FH10714** (a 7-aryl-substituted tubercidin), **FH8513** (a 7-aryl-substituted 3′-deoxytubercidin) and pentamidine (the positive control) were tested on the promastigotes of LmexCas9 LmexCas9^ΔNT1^ and LmexCas9^ΔNT1+TcrNT2^ (Table 5). In line with the Finley et al. report from 1988 [18], the present data confirmed that tubercidin is indeed a substrate for TcrNT2 as, when expressing this carrier in Cas9^ΔNT1^, the cells were over 30-fold sensitized to the analog, with an EC_50_ of 0.82 ± 0.13 µM (*p* < 0.001). **FH8513** displayed only marginal effects on Cas9 and Cas9^ΔNT1^, with EC_50_ values around 100 µM, with the highest concentration tested in the assay. The introduction of TcrNT2 significantly sensitized the cells to this compound, resulting in an EC_50_ of 31.1 ± 0.5 µM (*p* < 0.01), showing that (1) TcrNT2 mediates the uptake of **FH8513**, a 3′-deoxytubercidin analog and (2) that unmodified *L. mexicana* promastigotes are not sensitive to 3′-deoxytubercidins because of the limitations of trans-membrane transport. This explains, at least in part, why *T. cruzi* are more sensitive than *Leishmania* spp. to this class of compounds [20]. In contrast, the tubercidin analogs **FH11706** and **FH10714** had no effect on any of the three *L. mexicana* strains. Of note, both compounds feature fluoro substitutions on positions 3 and/or 4 of the 7-phenyl ring. When these were 3- and/or 4-chloro substitutions instead (**TH1012**, **FH3147**) or even an unsubstituted phenyl ring (**TH1004**), Cas9 was sensitive to the analogs, and the deletion of LmexNT1 caused high levels of resistance, which were mostly reversed upon the introduction of TcrNT2. Most clearly, LmexCas9 was highly sensitive to 7-(3,4-dichlorophenyl)tubercidin **FH3147** (EC_50_ 8.4 ± 1.6 µM) but became insensitive upon the deletion of LmexNT1 (EC_50_ > 100 µM), which was reversed upon the expression of TcrNT2 (EC_50_ 13.7 ± 1.3 µM). These observations show that TcrNT2 can transport 7-aryltubercidins, although this ability could not be demonstrated for **FH11706** and **FH10714**. The insensitivity of the *L. mexicana* promastigotes to these compounds is likely because of the inherent insensitivity of the cells to the fluorinated analogues rather than an inability of LmexNT1 or TcrNT2 to take up 7-aryltubercidins. Apart from the sensitivity data presented above and in Table 5, direct measurements of the affinity of tubercidin and some 7-substituted analogs for LmexNT1 were performed using [^3^H]-adenosine as the substrate. As shown in Figure 2, tubercidin showed high affinity for LmexNT1 (K_i_ 5.8 ± 0.6 µM; *n* = 3), and 7-substitution with phenyl or chloro, if anything, enhanced the affinity: **FH3169** K_i_ 2.8 ± 0.2 µM (*p* < 0.05) and **TH1004** K_i_ 3.8 ± 0.3 µM (*p* > 0.05). These data demonstrate that 7-aryl-substituted tubercidins are good substrates for both LmexNT1 and TcrNT2 but that *T. cruzi* is substantially more sensitive to this class of compounds than *L. mexicana*. 

### 2.6. In Vivo Analysis of the Derivatives in a Mouse Model of Acute Toxicity and T. cruzi Infection

The high activity and selectivity of the derivatives against all relevant parasite forms and DTUs of the parasite prompted us to check their efficacy in a mouse model of acute toxicity and *T. cruzi* infection. Then, proof-of-concept assays were carried out according to the guidelines established by the FIOCRUZ Committee of Ethics for the Use of Animals as described in Material and Methods.

**Figure 3:**
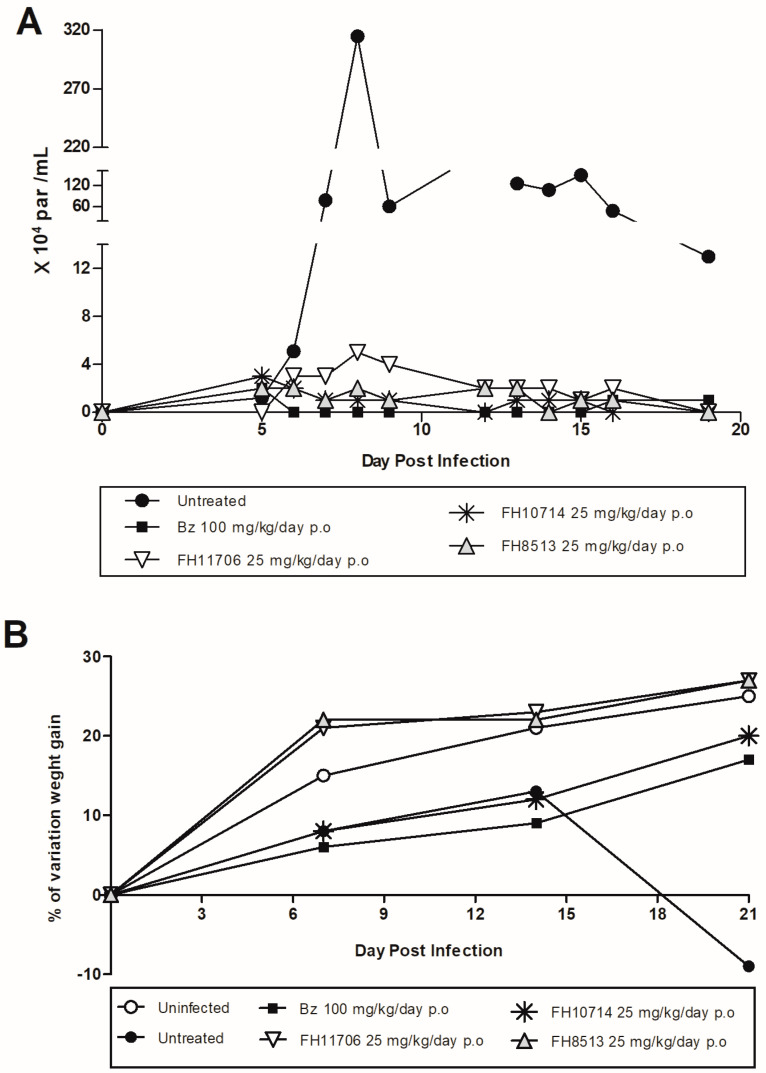
**FH11706**, **FH10714**, **FH8513** and Bz activity on male Swiss mice infected with 10^4^ bloodstream trypomastigotes (Y strain). (**A**) Parasitemia curve. (**B**) Percentage (%) of mouse weight variation.

All derivatives were given orally to male Swiss mice for five consecutive days at a dose of 50 mg/kg/day, following the suggested screening cascade for the identification and progression of new chemical entities for CD [31,32]. The mice were observed for 5 days for overt toxic events, but none were evident. Thus, a proof-of-concept study was performed in *T. cruzi*-infected mice. The infected animals were treated (gavage, once a day) for 5 consecutive days with the derivatives (25 mg/kg/day) or Bz (100 mg/kg/day), starting drug administration at the parasitemia onset, usually on day 5 dpi, following doses and protocols already established for the other nucleoside derivatives [22]. **FH11706**, **FH10714** and **FH8513** suppressed the parasitemia, resulting in reductions of 98.4, 99.58 and 99.48%, respectively (*p* < 0.05), similar to Bz (Figure 3A). The nucleoside analogues, particularly **FH11706** and **FH8513**, strongly protected the animals against the loss of weight induced by the acute experimental *T.cruzi* infection (Figure 3B).

The molecular analysis of the blood parasite load demonstrated that all compounds failed to provide a sterile cure since all treated animals displayed positive qPCR. At 77 dpi, blood samples were collected by heart puncture from animals treated with **FH11706**, **FH10714**, **FH8513** and Bz. In all groups, a low parasite DNA amplification was detected (0.038 ± 0.016, 0.046 ± 0.038, 0.287 ± 0.017 and 0.187 ± 0.082 eq par/mL, respectively), being below the limit of detection (0.47 eq par/mL). The vehicle-treated animals presented the highest parasite DNA amplification (1.17 ± 0.45 eq par/mL). 

### 2.7. In Vitro Washout Assays in T. cruzi-Infected L929 Cultures (Tulahuen Strain)

**Figure 4:**
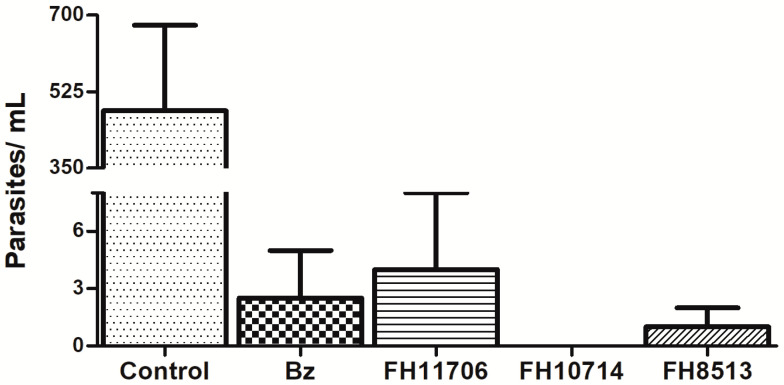
Light microscopy quantification of parasites released into the supernatant of L929 infected with *T. cruzi* (Tulahuen strain) after washout assay. Values are the average of two independent determinations. All compounds were statistically significative (*p* > 0.05) compared to untreated cultures (control).

To explore in vitro parasitic exhaustion, *T. cruzi*-infected L929 cultures were incubated for seven days with the compounds at 10 μM. Then, fresh drug-free medium was added and replaced every 48 h. After seven days of incubation, the number of released parasites into the supernatant was quantified by light microscopy. All of the derivatives reduced the number of released parasites, but only **FH10714** cleared the parasitism (Figure 4).

## 3. Discussion

As part of a previous broader screening of nucleoside analogs, three derivatives, named **FH11706**, **FH10714** and **FH8513,** were identified as promising anti-*T. cruzi* agents [19,20,21], and in the present study, they were further evaluated in vitro and in vivo. The findings corroborated the earlier reports regarding the high potency of tubercidin analogues, especially **FH8513**, which was about 10-fold more active than benznidazole when its intracellular forms (Tulahuen strain in L929 cell cultures) were assayed [19]. Also, **FH8513** displayed an outstanding effect against bloodstream forms, with EC_50_ value like Bz.

**FH11706**, **FH10714** and **FH8513** were low-to-moderate toxic to monolayers of different cell lines (fibroblasts L929 and cardiomyoblasts H9C2). As 3D structures may behave more closely to in vivo responses and are claimed as an important scientific tool to identify safer and more potent drugs [29,30], cardiac microtissues were evaluated. The H9C2 microtissues failed to detect cardiotoxicity for these tubercidins or for pentamidine, a well-known toxic drug [33]. It is possible that the cardiac spheroids present lower permeability to these compounds, as already reported for other drugs [34,35,36]. However, when the anti-*T. cruzi* activity of the derivatives was further investigated using the 2D and 3D cultures of H9C2 infected with the Y strain, the light microscopy and qPCR analysis revealed that all derivatives sustained high potency against the intracellular forms in both cultures, similarly as Bz. This shows that the nucleosides did penetrate well into the artificial tissue despite the lack of toxicity. 

In protozoa, all genes known to encode for nucleoside and nucleobase transporters are of the Equilibrative Nucleoside Transporter family [15,37], although there have been suggestions that some highly specific nucleobase carriers for uracil, adenine and hypoxanthine may belong to a different gene family but, if so, this other gene family has not yet been identified [38,39]. Recently, the four *T. cruzi* ENT genes were cloned and characterized, and the findings showed a specific adenine transporter (TcrNB2, [17]), a hypoxanthine/guanine transporter (TcrNB1), an inosine/guanosine transporter (TcrNT1) and a thymidine transporter (NT2) [16]. The observation of Finley (1988) [18] that tubercidin resistance in *T. cruzi* is associated with the loss of uptake of thymidine prompted us to test whether TcrNT2 can mediate the uptake of tubercidin and its analogues. Using the previously described *L. mexicana* Cas9^ΔNT1^ strain [17,40] to express TcrNT2, the present data demonstrated the sensitization of Cas9^ΔNT1+TcrNT2^ to tubercidin and multiple analogues, including 3′-deoxytubercidin analogue **FH8513** and 7-aryl and 7-halogen tubercidin analogues. **FH11706** and **FH10714** had no effect on any of the *L. mexicana* strains. This is most likely the result of *Leishmania* promastigotes simply not being sensitive to these 7-fluorophenyl-substituted analogues, as 7-substituted tubercidins were shown to be high-affinity substrates of LmexNT1. Thus, 7-substituted tubercidins, such as tubercidin itself, are most likely taken up by TcrNT2. This was demonstrated by the observation that the expression of TcrNT2 in LmexCas9^ΔNT1^ sensitized the cells highly significantly sensitized the cells to the multiple 7-substituted tubercidin analogs, **FH3169**, **TH1012** and **FH3147**.

The in vivo efficacy studies are in line with previous data reporting that despite the strong suppression of parasitemia, the nucleoside derivatives did not achieve a full sterile cure in a mouse model of a *T. cruzi* acute infection with the standardized oral administration protocols [19,20,21,22,41]. The lack of a sterile cure has been associated with the inactivity of the derivatives on the non-metabolically active and non-dividing trypomastigotes and/or the existence of dormant/persistent forms of the parasite [22,41]. Parasitic recrudescence has been studied in several models and is partly related to the dormant/persistent forms that may spontaneously arise and that can resume cell proliferation after removing the pressure induced by different stress factors and/or even voluntarily by mechanisms not yet understood [42,43]. The washout assays revealed that only **FH10714** eliminated the parasitism after seven days of incubation in the L929 monolayers. In fact, although **FH10714** appeared to eliminate the parasite from 3D cultures, it was incapable to promote a parasitological cure in vivo when given once a day, for 5 days at 25 mg/kg. Given the oral route of administration, it must be considered that suboptimal acid stability, intestinal absorption and chemical stability in vivo may limit the availability of the compound in the animal model, and these issues need urgent further investigation. 

Although not providing a full animal cure, qPCR values revealed that in vivo treatment using the nucleoside analogues resulted in very low levels of parasitism (consistent with observations using the 3D cultures), being lower than the CD reference drug. These findings argue in favor of additional studies of pharmacokinetics and/or longer periods of in vivo drug administration, as well as in combinatory protocols. Also, future analysis of an extended duration in vitro washout using a larger panel of *T. cruzi* strains may provide additional information to move new nucleoside analogues to animal studies since drug sensitivities may be related to differences in *T. cruzi* strain response and replication rates [44,45].

## 4. Conclusions

The present data report the in vitro potency of nucleoside derivatives against *T. cruzi*, with EC_50_ values inferior to Bz against intracellular forms and comparable against bloodstream forms. Their uptake appears to be mediated by the TcrNT2 nucleoside carrier. The compounds were highly active in vivo but failed to induce a complete parasitological cure when given orally for 5 days, justifying further studies with longer treatment regimens, as well as evaluation in combination with nitro drugs, aiming to identify more effective and safer therapies for Chagas disease.

## 5. Material and Methods

### 5.1. The Studied Compounds

The synthesis and characterization of **FH11706**, **FH10714** [46] and **FH8513** [19] were previously reported. Benznidazole (Bz) was purchased from Laboratório Farmacêutico do Estado de Pernambuco (Brazil). For in vitro analysis, stock solutions were prepared at 20 mM in 100% dimethyl sulfoxide (DMSO), and compounds serially diluted (2-fold) in RPMI culture medium, with the final DMSO concentration never exceeding 0.6%, which does not induce cellular damages to mammalian cells and *T. cruzi* parasites [47]. For in vivo assays, Bz was formulated using 3% Tween 80 in distilled water and the nucleoside analogues in 10% (*v*/*v*) EtOH, with 1.1 mM aqueous citrate buffer pH 3.2 and dosed according to animal body weight.

### 5.2. In Vitro Cultures of Mammalian Cells

L929 cell lines were used to evaluate the effect of the nucleoside derivatives on the cellular viability of mammalian host cells and to assess their activity against intracellular forms of *T. cruzi* (Tulahuen strain expressing *E. coli* β-galactosidase gene, DTU VI) as reported [27]. 2D and 3D culture systems of rat cardiomyoblast cell lines (H9C2(2-1)) were used to evaluate cardiotoxicity and to test the derivatives’ activity on intracellular forms (Y strain, DTU II) [48]. In all assays, the cell cultures were maintained at 37 °C under an atmosphere of 5% CO_2_.

### 5.3. The Obtention and Maintenance of the Parasites

Bloodstream trypomastigotes of the Y strain (DTU II) were obtained by cardiac puncture of infected Swiss Webster mice on the parasitemia peak to perform drug sensitivity assays [49]. Trypomastigote forms of the Tulahuen-β gal strain were collected from the supernatant of previously infected L929 cell cultures (host/parasite cell ratio 10:1) [22]. The intracellular forms *T. cruzi* were studied by the infection of L929 and H9C2 (2D and 3D) cell cultures infected for 48 and 24 h with trypomastigotes of Tulahuen-β gal and Y strains, respectively [50]. The *Leishmania mexicana*-Cas9 T7 strain (LmexCas9) was derived from *L. mexicana* WT promastigotes by expression of the *Streptococcus pyogenes* Cas9 nuclease gene ‘Cas9′ and maintained on 32 μg/mL hygromycin [51]. This strain and the derived strains Cas9^ΔNT1^ and Cas9^ΔNT1+TcrNT2^ were cultured in HOMEM medium supplemented with 10% of FBS and 1% Penicillin-Streptomycin antibiotic in unvented flasks at 25 °C [52]. The Cas9^ΔNT1^ strain is identical to Cas9 except that the LmexNT1.1/NT1.2 locus encoding the *L. mexicana* adenosine/ pyrimidine transporters was excised using Cas9 [17,40]. Cas9^ΔNT1+TcrNT2^ is the further modification of that strain by the expression of the *T. cruzi* nucleoside transporter 2 (TcrNT2) gene in it [17], using the vector pNUS-HcN [53]. Appropriate antibiotics were added for the selection and maintenance of transfectants.

### 5.4. The In Vitro Toxicity on Mammalian Cell Cultures

L929 cells seeded in sterile 96-well microtiter plates were exposed or not to increasing concentrations (up to 200 µM) of the compounds. After seven days of incubation, the cell growth of treated cultures was compared to untreated control cells (100% cell growth). Cellular viability was assessed through AlamarBlue^®^ (resazurin sodium salt) colorimetric assay, and the results were expressed as % reduction in cell growth/viability compared to untreated control, and an IC_50_ value determined [27]. For the assays using H9C2 cultures, 25 × 10^3^ cells/well were seeded in a 96-well microtiter plates (flat and U bottoms, precoated with agarose 1% for preparing 2- and 3D matrices, respectively) and maintained at 37 °C [48,54].

The cardiac cultures were exposed or not to increasing concentrations of the nucleoside derivatives (up to 300 µM), and after seven days of incubation, their physiology and morphology was evaluated by light microscopy. Cell viability was measured using the PrestoBlue^®^ (PB) colorimetric assay, the results evaluated as % reduction in cell growth/viability compared to untreated control, and an IC_50_ value determined [54]. The data results from at least two independent experiments done in at least duplicates.

### 5.5. The In Vitro Activity of the Compounds against Intracellular Forms of T. cruzi (Tulahuen and Y strains) in L929 and H9C2 Monolayers, Respectively

In an initial step, nucleoside derivatives were assayed against intracellular forms of Tulahuen-β gal strain in L929 cell lines [27]. Assays were performed in sterile 96-well microtiter plates with 4 × 10^3^ cells/well and 4 × 10^4^ parasites/well (host/parasite cell ratio 1:10). After the establishment of infection (48 h), increasing concentrations (up to 30 µM) of the compounds were added, and the cultures maintained at 37 °C for seven days. Untreated-infected controls represented 100% growth. Parasite burden was analyzed by adding the substrate CPRG (chlorophenol red ß-D-galactopyranoside) followed by measurements (spectrophotometrically) at 570 nm. The results were expressed as percentage of reduction in parasite growth compared to untreated cells, and EC_50_ values were calculated. Bz was used as a positive control [27,55]. To investigate the effect of the compounds against intracellular forms of the Y strain, rat cardiomyoblasts (2D cultures of H9C2) were seeded in 24-well plates and, after 24 h of platting, were infected with bloodstream trypomastigotes (20 parasites per host cell). After 24 h of interaction, the infected cultures were rinsed to remove non-internalized parasites and incubated with 10 µM of the tested compounds. After seven days of treatment, the cultures were fixed, stained with Giemsa, and 200 cells were quantified by light microscopy. The results were expressed as % of reduction in parasite burden [56]. The data results from two independent experiments performed in at least duplicate were obtained.

### 5.6. The In Vitro Activity of the Compounds against Intracellular Forms of T. cruzi (Y strain) in H9C2 Organoids

3D cultures of H9C2 cells were obtained as described [54], and 24 h after plating, the spheroids were infected (or not, control) with bloodstream trypomastigotes of *T. cruzi* (strain Y), using 20 parasites per host cell. After 24 h, infected spheroids were incubated with 10 µM of the tested compounds for seven days, and then the spheroids were transferred one by one to an Eppendorf tube, with the aid of a pestle they were mechanically macerated following an established protocol [57] with minor adaptations. The parasite load was quantified by light microscopy [55] and qPCR [54], and the results were expressed as % of parasite death. The data results from two independent experiments performed in at least duplicate. 

### 5.7. The In Vitro Activity of the Compounds against Bloodstream Forms of T. cruzi (Y strain)

To analyze the compound activity against bloodstream trypomastigotes of *T. cruzi* (Y strain), 5 × 10^6^ mL of parasite were added in 96-well microtiter plates and incubated for 2 or for 24 h at 37 °C in RPMI culture medium in the presence or absence of each compound (up to 30 µM). The death rates were quantified by light microscopy using a Neubauer chamber to determine the EC_50_ [49,58]. The data results from two independent experiments performed in duplicate were obtained. 

### 5.8. The In Vitro Activity of the Compounds against Leishmania mexicana Promastigotes

The EC_50_ values for nucleoside analogues against *Leishmania mexicana* promastigotes of the Cas9, Cas9^ΔNT1^ and Cas9^ΔNT1+TcrNT2^ strains used a resazurin-based assay, as described [59]. Briefly, the assays were performed in standard HOMEM medium and culture conditions, in white opaque 96-well plates. For each test compound (stored as a 40 mM stock solution in 100% DMSO at −20 °C), a doubling dilution was performed over 2 rows of the plate, keeping the last well free as no-drug control with HOMEM only (23 doubling dilutions; highest concentration 100 µM). To each well, 100 µL of HOMEM containing 2 × 10^5^ cells of the appropriate *L. mexicana* line was added, and the plates incubated for 72 h at 25 °C, followed by the addition of 20 µL of resazurin solution and further incubation for 48 h. Fluorescence was read on a FLUOstar OPTIMA plate reader (BMG Labtech, Germany), at an excitation wavelength of 544 nm and an emission wavelength of 620 nm. The EC_50_ values were determined by the GraphPad Prism 8.0 software using an equation for a sigmoid curve with variable slope. Pentamidine was used as positive control, and every experiment was independently performed at least 3 times independently.

### 5.9. In Vitro Analysis of Derivatives Uptake by TcrNT2 Thymidine Transporter

Uptake of 50 nM 2,8-^3^H-adenosine (American Radiolabelled Chemicals (ART0287A), 40 Ci/ mmol) was exactly measured, exactly as described [26]. Briefly, promastigotes were washed into the standard assay buffer (AB) by centrifugation, and aliquots of 100 µL containing 10^7^ parasites were incubated with the radiolabel on top of an oil layer in a microfuge tube, for exactly 5 s. The incubation was stopped by the addition of 800 µL of ice-cold 1 mM adenosine in AB and immediate centrifugation through the oil layer to separate cells from the remaining extracellular radiolabel. Cell pellets were collected in scintillation vials after flash freezing in liquid nitrogen, by cutting of the tip of the microfuge tube. The promastigotes were then solubilised in 1% SDS, and scintillation fluid was added (Scintlogic U, Lablogic), and radioactivity was quantified in a Hidex 300SL scintillation counter (Lablogic). Data was plotted using non-linear regression with an equation for a sigmoid curve with a variable slope in Prism v8 (GraphPad). 

### 5.10. In Vitro Washout Assays in T. cruzi-Infected L929 Cultures (Tulahuen Strain)

*T. cruzi*-infected L929 cultures (using the Tulahuen-β gal strain) were exposed or not to 10 µM of each nucleoside derivative, and the RPMI culture medium (containing the tested compounds) replaced every 48 h. After seven days of incubation, the cultures were rinsed with 0.1 M phosphate buffered saline (PBS), and drug-free culture medium was added for another seven days of incubation, replacing the drug-free medium every 48 h. Parasites released into the supernatant of the cell cultures were quantified using light microscopy, and the parasite burden into the infected cultures was analyzed by adding the substrate CPRG (chlorophenol red ß-D-galactopyranoside) followed by spectrophotometric measurements at 570 nm. The results were expressed as percentage of reduction in parasite growth compared to untreated cells, and EC_50_ values were calculated. Bz was used as a positive control [27,55]. The data results from two independent experiments done in at least duplicate.

### 5.11. In Vivo Analysis of the Derivatives in a Mouse Model of Acute Toxicity and T. cruzi Infection

Male Swiss Webster mice (18−20 g; 4−5 weeks of age) were obtained from the Institute of Sciences and Technologies in Biomodels (ICTB-FIOCRUZ), housed with a maximum of 5 animals per cage and kept in a specific pathogen-free (SPF) room at 20−24 °C under a 12 h light and 12 h dark cycle. All animals received sterilized water and food ad libitum. The animals were acclimatized for 7 days before the experiments. To detect possible toxic events, nucleoside analogues and Bz were orally given to non-infected Swiss male mice (*n* = 2) for 5 consecutive days, with a fixed dose of 50 mg/kg (0.1 mL). On day 5, all animals were euthanized to inspect toxic and subtoxic symptoms [49]. For a proof-of-concept study, animals were infected by i.p. administration of 10^4^ bloodstream trypomastigotes (Y strain), and drug administration started at the onset of parasitemia (6 dpi), only using mice with detectable parasitemia. Control mice groups were age-matched and housed under identical conditions. The following experimental groups were used (3 mice per group): untreated (infected vehicle-treated control) and treated (infected and treated with derivatives or with benznidazole). The derivatives and Bz were given orally (gavage) for five consecutive days at 25 mg/kg and 100 mg/kg, respectively, once a day. Parasitemia levels in *T. cruzi* assays were individually checked by light microscopic counting of parasites in 5 μL of blood, as described [22]. For blood qPCR analysis, 500 µL of blood was diluted in a 1:2 volume of guanidine solution and heated for 60 s in boiling water, followed by DNA purification using High Pure PCR Template Preparation Kit (Roche Applied Science) and quantitative real-time multiplex PCR assays. TaqMan probes were used for quantification of both *T. cruzi* satellite nuclear DNA and internal amplification control (IAC), as described in Duffy et al. (2013) [60], using the following primer and prober sets: Cruzi1 5′-ASTCGGCTGATCGTTTTCGA-3′ (750 nM), Cruzi2 5′-AATTCCTCCAAGCAGCGGATA-3′ (750 nM), Cruzi3 FAM-5′-CACACACTGGACACCAA-3′-NFQ-MGB (50 nM), IAC Fw 5′-ACCGTCATGGAACAGCACGTA-3′ (100 nM), IAC Rv 5′-CTCCCGCAACAAACCCTATAAAT-3′ (100 nM) and IAC Tq 5′-VIC-AGCATCTGTTCTTGAAGGT-NFQ-MGB-3′ (50 nM). The cycling conditions were a first step of 10 min at 95 °C, followed by 40 cycles at 95 °C for 15 s and 58 °C for 1 min. The amplifications were carried out in a Quantstudio 3 real-time PCR system (Applied Biosystems), with the threshold set at 0.02 in all runs. Standard curves were constructed for absolute quantification through the serial dilution of total DNA, ranging from 10^5^ parasite equivalent to 1 parasite equivalent per mL of blood, obtained with a negative blood sample in guanidine-EDTA. Parasite load was expressed as equivalent of parasite DNA per mL of blood [61].

### 5.12. Statistical Analysis

The analyses were obtained through nonlinear regression analysis by GraphPad Prism v 9.0 (GraphPad Software, San Diego, CA, USA). Statistical analysis was performed using an ANOVA single factor test with the level of significance set at *p* ≤ 0.05.

### 5.13. Ethics

All animal studies were carried out in strict accordance with the guidelines established by the FIOCRUZ Committee of Ethics for the Use of Animals (CEUA L038-2017).

## Figures and Tables

**Table 1:** Activity of the studied compounds against intracellular forms of *T. cruzi* (Tulahuen strain), toxicity on L929 cells and selective indexes (SI) after seven days of compound incubation.

Compound	Activity on Intracellular Forms		Toxicity on L929 IC_50_, µM	SI *
	(EC_50_, µM)	(EC_90,_ µM)		
**Bz**	1.97 (1.36 to 2.86)	5.54 (2.66 to 8.42)	>200	>101.5
**FH11706** (**1**)	1.11 (0.62 to 1.97)	>30	118.5 (75.31 to 186.4)	106.7
**FH10714** (**2**)	0.47 (0.24 to 0.93)	6.19 (0.58 to 12.96)	55.75 (48.37 to 64.25)	118.6
**FH8513** (**3**)	0.20 (0.10 to 0.41)	1.61 (0.32 to 2.90)	22.27 (13.63 to 36.38)	111.3

* SI represent the ratio of IC_50_ and EC_50_ values (IC_50_/EC_50_). Data represent the average of three independent determinations; 95% of confidence intervals are indicated.

**Table 2:** Activity of the studied compounds against bloodstream trypomastigotes of *T. cruzi* (Y strain), toxicity on H9C2 and respective selective indexes (SI) after 24 h of compound incubation.

Compound	Activity upon Bloodstream Forms		H9C2 IC_50_ µM	SI *
	EC_50_, µM	EC_90_, µM		
**Bz**	6.9 (3.9 to 12.5)	19.62 (11.5 to 33.5)	>300	>43.48
**FH11706** (**1**)	17.58 (12.7 to 24.4)	>30	>300	>17.06
**FH10714** (**2**)	13.40 (9.1 to 19.7)	>30	>300	>22.39
**FH8513** (**3**)	5.9 (3.8 to 9.3)	16.73 (5.3 to 53)	267.5 (235.1 to 304.4)	45.34

* SI represents the ratio of the IC_50_ and the EC_50_ (IC_50_/EC_50_). Values are the average of three independent determinations; 95% of confidence intervals are indicated.

**Table 3:** Cardiotoxic profile (IC_50_) of the studied compounds using 2D and 3D cultures of H9C2 after seven days of compound incubation.

Compounds	IC_50_ (µM)	
	2D	3D
**Bz**	>200	>200
**Pt**	45.98 (15.39 to 137.4)	>200
**FH11706** (**1**)	47.71 (32.62 to 69.79)	>200
**FH10714** (**2**)	41.82 (25.23 to 69.30)	180.8 (153.6 to 208)
**FH8513** (**3**)	18.57 (11.36 to 30.36)	199.8 (120.4 to 331.4)

Values are the average of three independent determinations; 95% of confidence intervals are indicated.

**Table 4:** Activity of the studied compounds (10 µM, seven days of incubation) against *T. cruzi* (Y strain) infection of H9C2 (monolayers and spheroids). The parasite load was determined by light microscopy and qPCR quantification.

Compound	% of Death Rate by Light Microscopy Quantification		% of Death Rate by qPCR 3D
	2D	3D	
**Bz**	100	98.4 (98.1 to 98.7)	100
**FH11706** (**1**)	99.25 (98.83 to 99.67)	91.6 (86.2 to 97)	85 (79 to 91)
**FH10714** (**2**)	100	93.5 (93.46 to 93.54)	67 (38 to 96)
**FH8513** (**3**)	99.14 (97.9 to 100)	83.8 (67.5 to 100)	94 (90 to 98)

Values are the average of two independent determinations; 95% of confidence intervals are indicated.

**Table 5:** Effects of tubercidin and its analogues on promastigotes of the LmexCas9, Lmex^ΔNT1^ and Lmex^ΔNT1+TcrNT2^ strains.

	Cas9	Cas9^ΔNT1^	Lmex^ΔNT1+TcrNT2^			
	EC_50_ (μM)	EC_50_ (μM)	EC_50_ (μM)	Sensitization (fold)	*p* vs. Cas9^ΔNT1^	
Tubercidin	1.02 ± 0.16	25.7 ± 0.94	0.82 ± 0.13	31.2	<0.001	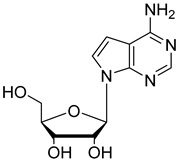
**1** (**FH11706**)	>100	>100	ND			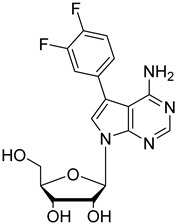
**2** (**FH10714**)	>100	>100	>100			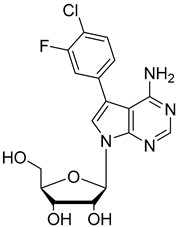
TH1012	52.5 ± 0.3	280 ± 18	96.6 ± 0.3	2.9	<0.001	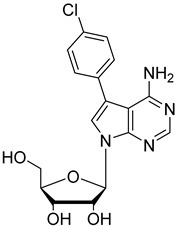
FH3147	8.41 ± 1.61	>100	13.7 ± 1.25	7.3	<0.001	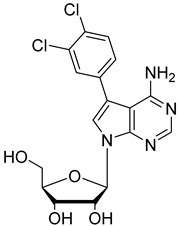
FH3169	0.24 ± 0.06	2.00 ± 0.19	0.32 ± 0.05	6.3	<0.01	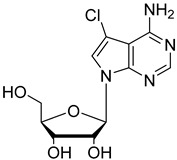
TH1004	37.0 ± 0.23	138 ± 10	101 ± 0.5	1.37	<0.05	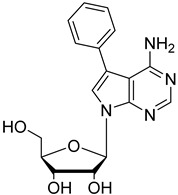
**3** (**FH8513**)	>100	100.5 ± 0.9	31.1 ± 0.5	3.2	<0.01	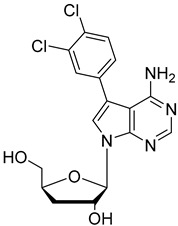
Pentamidine	0.87 ± 0.10	1.25 ± 0.22	1.12 ± 0.17	1.11	>0.05	

EC_50_, 50% effective concentration determined in a resazurin-based assay; sensitization is determined as the ration of the EC_50_ values of the Cas9^ΔNT1^ and Cas9^ΔNT1+TcrNT2^ strains. The *p* value is based on an unpaired, two-tailed Student’s t-test comparing the EC_50_ values of the same two strains (*n* ≥ 3).

## Data Availability

All relevant data are included in the manuscript.

## Sample Availability

All relevant data are included in the manuscript.
